# Effect of ultrasonication on the stability and storage of a soy protein isolate-phosphatidylcholine nanoemulsions

**DOI:** 10.1038/s41598-020-70462-8

**Published:** 2020-08-19

**Authors:** Fei Teng, Mingyu He, Jingwen Xu, Fanfan Chen, Changling Wu, Zhongjiang Wang, Yang Li

**Affiliations:** 1 College of Food Science, Northeast Agricultural University, Harbin, 150030 Heilongjiang China; 2 Harbin Institute of Food Industry, Harbin, 150030 Heilongjiang China; 3 Heilongjiang Academy of Green Food Science, Harbin, 150030 Heilongjiang China

**Keywords:** Nanoparticles, Nanostructures

## Abstract

Phosphatidylcholine-soybean protein isolate (PC-SPI) nanoemulsions were prepared by ultrasonication. The effects of preparation conditions (SPI and PC addition, ultrasonic power and time) on the structural properties of the nanoemulsions and their storage stability were investigated. The results showed that the most optimal adsorption capacity and adsorption tightness at the oil–water interface under optimal conditions (1.5% SPI, 0.20% PC, 500 W ultrasonic power and 9 min ultrasonic time) were exhibited by the SPI-PC conjugate, which demonstrated that this nanoemulsions can be categorized as a high-quality emulsion suitable for research. To test its stability, and the high-quality nanoemulsion of β-carotene was stored. After degradation of the nanoemulsions during storage, β-carotene was released. The β-carotene retention rate of the high-quality emulsion was maintained above 86% at different temperatures in the absence of light for up to 30 days. This study provides new information for the development of transport and stability systems for nanoemulsions.

## Introduction

Nanoemulsions are carriers with strong kinetic stability and bioavailability that are used to transport lipophilic substances^1^. Compared with conventional emulsions, nanoemulsions with particle sizes of mainly 100–500 nm have higher stability, solubility, bioavailability, and transport efficiency^2^. Nanoemulsions can increase the availability of biologically active substances in specific parts via controlled release^3^. Nanoemulsions are usually prepared using a high-energy (mechanically based) or low-energy (chemically based) method. High energy technologies such as homogenization, microfluidization and ultrasound technology have been used to prepare nanoemulsions^4^. However, microfluidic and homogenization technologies were not readily available in industry due to high costs and scalability issues^5^. Ultrasonic processing has the advantages of easy operation of the system, low production cost, low requirements for instruments and high energy efficiency^6^. Hydraulic shear and cavitation are produced by ultrasonication to break droplets to nanodroplets^7,8^.

Soybean protein isolate (SPI) is widely used in food formulations due to its nutritional value and functional characteristics^9^. SPI as an emulsifier plays a vital role in the formation of small oil droplets. The introduction of external emulsifiers (such as lecithin) can enhance the emulsification properties of SPI in producing nanoemulsions^10^. Phosphatidylcholine (PC) is the main phospholipid in lecithin^11^. Lecithin contains a lipophilic group in the form of a fatty acid group and a hydrophilic group in the form of a phosphate. The amphiphilic molecular structure is responsible for the excellent emulsification ability of PC, and the physical stability of an SPI nanoemulsion can be increased by adding lecithin^12,13^. Emulsifiability increases when PC interacts with SPI through a hydrophobic group. Li et al.^14^ proved that a binding site can be a hydrophobic region of the α-helix of a protein.

β-Carotene (C40H56) is a natural pigment that is found in various fruits, grains, and oils. It is a lipophilic substance and a highly conjugated long-chain isoprenoid micronutrient^15^. β-Carotene has been widely used in food, feed, and other nutraceuticals as an antioxidant^16^. Some functionalities of β-carotene are limited because its lower chemical stability of carotenoids, which is easily degraded by heat, oxygen, and light. Because the solubility of β-carotene in some common solvents, such as water, dimethyl sulfoxide (DMSO), and ethanol, is very low, it is difficult to add it to foods to maintain its stability^17^. β-Carotene can be dissolved in oil at 25 °C, and the bioavailability of β-carotene can be increased by combining it with digestible lipids in the human body^16^. In addition, the biological activity of β-carotene against heart disease, cataracts, and cancer has been previously demonstrated^18^. Therefore, the emergence of nanoemulsions provide a new way for the successful transportation of β-carotene.

Emulsification, aggregation, flocculation, and precipitation may occur in emulsions due to unstable thermodynamic systems^19^. Nanoemulsions that are physicochemically stable can provide some ζ-potential advantages that can be used in the development of food. Temperature, pH, storage time, processing method, and ionic strength influence the stability of emulsions^20^. The main factors affecting emulsion stability were temperature and time during storage. In this research, ultrasound was used to prepare soybean protein-phosphatidylcholine nanoemulsion, and β-carotene was embedded into nanoemulsion to improve the stability and bioavailability of β-carotene. After preparing the nanoemulsion, the best preparation process of β-carotene nanoemulsion was determined. In addition, their stability was checked, and the effect of different storage temperature and storage time on the stability of the nanoemulsion was determined to ensure β-carotene. The stability of nanoemulsion during storage and transportation to expand its application in food processing.

## Materials and methods

### Materials

SPI and PC (Liao Cheng, Shandong, China) were obtained from Gao Tang Co. Ltd., and sunflower oil (Duoli Group Co., Ltd., Shanghai, China) was obtained from a local supermarket. β-Carotene (≥ 97.0%) and acetone (insoluble content > 95%) were purchased from Sigma-Aldrich (St. Louis, MO, USA). Other compounds (analytical reagent grade) were obtained from the Kemiou Chemical Reagent Co., Ltd (Tianjing, China).

### β-Carotene nanoemulsion preparation

SPI (0.5%,1.0%, 1.5%, 2.0%, 2.5%, 3.0%) was mixed with PC (0.13%, 0.15%, 0.17%, 0.19%, 0.21%, 0.23%) with varying ratios before being dispersed in phosphate buffer solution (10.0 mM phosphate buffer, pH 7.5, 0.01% (w/w) sodium azide) followed by mixing at room temperature (25 °C) for 30 min using magnetic stirring^11^. β-Carotene (≥ 97.0%, 0.01 g, *w/w*) and sunflower oil (5 g, w/w) were mixed and magnetically stirred at room temperature for 30 min under the same conditions. The water and oil phase were homogenized with an Ultra-Turrax T18 homogenizer (Angni Co. Ltd., Shanghai, China) at room temperature at 20,000 rpm for 5 min to form a coarse emulsion. Ultrasonic treatment used a titanium probe with a diameter of 0.636 cm for ultrasonic cell disruption (Washin Instrument Co. Ltd., Wuxi, Jiangsu, China). The coarse emulsion was poured into a beaker surrounded by a double-walled cooling water jacket to minimize increases in temperature. Ultrasonic treatment at a frequency of 20 kHz and various output powers (200, 300, 400, 500 or 600 W), various treatment times (6, 7, 8, 9 or 10 min), pulse duration 4 s, off time 2 s . The freshly-prepared (O/W) emulsion was used for the following studies.

### Particle size, ζ-potential, and polydispersity index (PDI) analysis

The particle size, ζ-potential and PDI of nanoemuslions were measured using a dynamic light-scattering device (Zetasizer Nano-ZS 90 light scattering particle size analyzer). Nanoemulsions were diluted (1:1,000) with a phosphate buffer before measurement to prevent multiple scattering^21^. Aqueous dispersion and the refractive index medium were set at 1.33 and 1.45, respectively. The fresh nanoemulsions and phosphate buffer were diluted in proportion (1:100, *v/v*) during ζ-potential analysis.

### Turbidity analysis

Turbidity values were obtained using a previously published method with slight modification^22^. The nanoemulsions were diluted 40 times with a phosphate buffer solution. The absorbance was measured at 600 nm with a spectrophotometer (LW-1600FC UV, Shanghai, China). Phosphate buffer solution was used as a blank. Turbidity was calculated using the formula:

1 $$ T = 2.302 \times \frac{A \times V}{I} $$

where *A* = the absorbance of diluted nanoemulsions at 600 nm, *I* = optical path extent (0.01 m), and *V* = dilution (40).

### Emulsification yield analysis

The emulsification yield of the nanoemulsions was determined using a previously published method^11^. The β-carotene in 1 mL of the nanoemulsions was extracted using an organic phase (ethanol: n-hexane = 1:1, 95%, 5 mL) and was quantitated using an Agilent 1,100 HPLC (Agilent Technologies Inc., Santa Clara, CA, USA) system with a UV–Vis diode array absorption detector. A reversed phase analytical column C30 (YMC Carotenoid, 250 × 4.6 mm i.d., 5 μm, YMC, Inc., Wilmington, NC, USA) was used with a flow rate of 1.0 mL/min by mobile phase. A 20-μL sample was analyzed using a diode array detector (DAD) at 450 nm. Standard curves were obtained using different concentrations of β-carotene standard samples (assuming a purity of 100%). The amount of β-carotene in the nanoemulsions was determined after processing the data with Dionex Chromeleon software. The emulsion yield (%) of the nanoemulsions was calculated by the following formula:

2 $$ Emulsification\;yield = \frac{\beta - carotene\;content\;in\;nanoemulsion\;}{{total\;\beta - carotene\;content}} \times 100 $$

### Nanoemulsion stability analysis

Nanoemulsion stability was determined with a Turbiscan optical scanning analyzer (France Formulaction Company, Toulouse, France) according on the variation in the backscattering light intensity at different locations of the test tube using a previously published method with slight modification^23^. The analyzer was equipped with two detectors and a pulsed near-infrared light source (λ = 880 nm). The measurement process mainly used the central portion of the reflected spot, whereby the particle volume concentration and average particle size were directly related to the measured reflected light. Next, 20 mL of different loadings of β-carotene samples was placed in a cylindrical glass cell and then analyzed in the Turbiscan with scanning (from the bottom to the top) every 30 min for 6 h at 30 °C. The dynamic change in the reflected amount of light (ΔBS) indicated the stability of the SPI-PC nanoemulsions. The Turbiscan Stability Index (TSI) was calculated using the formula:

3 $$ TSI = \sqrt {\frac{{\sum\nolimits_{i = 1}^{n} {(x_{i} - x_{BS} )} }}{n - 1}} $$

where *x*_*i*_ = the average scattering intensity per minute, *x*_*BS*_ = the average value of *x*_*i*_, and *n* = the number of scans.

### Raman microscopic observation of nanoemulsion morphology

Raman microscopic observation was used as an interface structural analysis of the nanoemulsions^20^. A 5 mW laser was focused on a slide with droplets in the center, and the droplets were observed. The scanning distance was set in the direction of the two axes (x-axis, y-axis) according to the required scanning range. Spectral analysis was performed using Spec 32 software, which was supplied with the spectrometer. The moving distance of the spot was 2 μm, which enabled automatic and efficient scanning of the nanoemulsion sample to be tested. The 3D imaging portion observed by the microscope was based on 2D imaging. The laser focusing depth (i.e., z-axis) was adjusted so that layer-by-layer scanning of the sample could be manually conducted.

### Nanoemulsion storage stability

The β-carotene nanoemulsions were prepared with 1.5% SPI, 0.17% PC, ultrasonic power 500 W, and ultrasonic time 9 min. The storage stability of the β-carotene nanoemulsions was evaluated by adding 1 mL of the emulsion to a brown tube (1.5 mL) that was stored at 4, 25, or 55°C^24^. Next, 1 mL sample was extracted with 5 mL of n-hexane: ethanol = 3:2, and the hexane phase was removed. After repeating twice, their absorbances were measured using a spectrophotometer at 450 nm. A standard curve of β-carotene was prepared under similar conditions, and the β-carotene concentration was measured. The β-carotene retention rate was calculated using the formula:

4 $$ R_{\beta - carotene} = \frac{{C_{N} }}{{C_{I} }} \times 100\% $$

where *R*_*β-carotene*_ denotes β-carotene retention, *C*_*N*_ indicates the concentration of β-carotene in the nanoemulsions, and *C*_*I*_ represents the initial concentration of β-carotene.

### Statistical analysis

All measurements were analyzed by t-test via SPSS 16 software, and the results are expressed as the mean ± standard deviation (SD). Analysis of variance (ANOVA) was used to determine differences between groups.

## Results

### Effect of SPI addition

The effect of SPI addition on the characteristics and stability of nanoemulsions is shown in Table 1. The average particle size, PDI, turbidity, and TSI of the nanoemulsions were drastically decreased with the SPI content increased from 0.5% to 1.5%. The particle size value, turbidity, TSI and PDI value were minimized when the SPI addition was 1.5%. Hebishy found that when the whey protein content was low, the prepared nanoemulsions had larger droplets, which was consistent with the current results^25^. SPI as an emulsifier plays a vital role in the formation of small oil droplets by reducing the tension at the oil–water interface. When SPI is adsorbed on the surface of oil droplets, it prevents the oil droplets from accumulating and coalescing^26^.The thickness of the interfacial layer gradually increased and the interfacial tension at the oil–water interface decreased with SPI concentration, which increased the stability of the emulsion. High interfacial tension caused the emulsion droplets to fuse and aggregate with each other^27^.The maximum concentration of SPI adsorped at the interface is the critical micelle concentration. If the amount of SPI adsorption at the interface exceeded a critical value, the adsorption capacity of the protein molecule was weakened at the interface of the continuous phase.

**Table 1:** Effect of the soybean protein content on the characteristics and stability of nanoemulsions.

SPI addition	Particle size (nm)	ζ-potential (mV)	PDI	Emulsification yield (%)	Turbidity	TSI
0.5	797.0 ± 7.8^a^	− 28.90 ± 0.27^d^	0.56 ± 0.01^b^	69.11 ± 0.62^f^	26,758.4 ± 256.7^a^	5.50 ± 0.05^a^
1.0	606.0 ± 5.9^b^	− 29.50 ± 0.28^c^	0.44 ± 0.01^d^	78.20 ± 0.71^c^	21,150.8 ± 201.1^b^	4.30 ± 0.04^b^
1.5	295.6 ± 2.8^f^	− 33.40 ± 0.32^a^	0.21 ± 0.01^f^	90.03 ± 0.83^a^	14,896.6 ± 139.9^e^	3.21 ± 0.03^e^
2.0	382.3 ± 5.6^e^	− 33.30 ± 0.32^a^	0.37 ± 0.01^e^	81.25 ± 0.74^b^	18,561.0 ± 175.5^d^	3.41 ± 0.03^d^
2.5	471.5 ± 4.6^d^	− 30.50 ± 0.29^b^	0.52 ± 0.01^c^	76.48 ± 0.69^d^	19,420.3 ± 1836.4^bc^	3.90 ± 0.04^c^
3.0	549.0 ± 5.5^c^	− 29.60 ± 0.28^c^	0.66 ± 0.01^a^	70.02 ± 0.63^e^	20,837.7 ± 198.8^b^	4.21 ± 0.04^b^

Data represent mean ± standard deviations. Different letters in the same column represent a significant difference between samples (*p* < 0.05). Arrange all the averages in descending order, and use the letter “a” on the maximum average.

The stability of the nanoemulsion was positively correlated with its ζ-potential^11^. Table 1 showed that the absolute value of the ζ-potential was greater than 30 mV between 1.5% SPI and 2.5% SPI. It was observed that the ζ-potential values had a maximal value (-33.4 mV) at 1.5% SPI. The nanoemulsions exhibited satisfactory stability through electrostatic repulsion above 30 mV of the ζ-potential value^28^. It could be deduced that the nanoemuslions stabilized by SPI-PC complex had a high stability. With increasing addition of protein, the unabsorbed soluble protein formed aggregates under the action of ultrasonic cavitation and thermal effects due to enhanced hydrophobic interactions^20^. The protein at the oil–water interface was replaced by aggregates, leading to a decrease in emulsion stability^29^. Ghosh^5^ found that water molecules were difficult to penetrate the protein molecules under highly protein concentration. Protein molecules were aggregated, which decreased the stability of the nanoemulsions. Therefore, the emulsion yield value and the TSI value of the nanoemulsions were reduced.

### Effect of PC addition

PC is a surfactant with excellent biocompatibility and amphiphilicity, and it can increase the load capacity and reduce the interfacial tension of the dispersed phase by forming an embedded structure at the water/oil interface^30^. PC interacts with soy protein through electrostatic and hydrophobic interactions, and significantly affects the functional properties of soy protein^31^. The hydrophobic region of SPI can bind to the hydrophobic group of PC, and the binding force will maintain the interaction between SPI and PC^14,32^. Thus, The particle size, PDI value, TSI and turbidity of SPI-PC nanoemuslions were increased with the addition of PC from 0.13% to 0.17% as shown in Table 2. The smaller droplets were formed when the PC was increased due to sufficient protein isolate and insufficient interfacial tension to maintain newly formed droplets^7^ and thus, the stability of SPI-PC nanoemuslion was enhanced by adding more PC. This mixture system lowered the interfacial tension and also prevented coalescence, and thus improved the stability of a protein-stabilized oil–water emulsion^33^. Moreover, the soy protein molecule was negatively charged at neutral pH, while PC was a zwitterion with both positive and negative charges^34^. The addition of PC could increase the interface adsorption by enhancing the electrostatic interaction between hydrophobic SPI and PC. In the case of the emulsification interface, the blank palce found in soy protein was subsequently filled with PC.

**Table 2:** Effect of the lecithin content on the characteristics and stability of nanoemulsions.

PC addition	Particle size (nm)	ζ-potential (mV)	PDI	Emulsification yield (%)	Turbidity	TSI
0.13	604.5 ± 5.6^a^	− 26.90 ± 0.26^f^	0.73 ± 0.01^a^	88.11 ± 0.83^c^	26,311.0 ± 253.0^a^	5.00 ± 0.04^a^
0.15	470.2 ± 4.1^c^	− 28.20 ± 0.27^e^	0.60 ± 0.01^b^	88.90 ± 0.84^b^	23,986.8 ± 229.0^d^	3.91 ± 0.03^b^
0.17	295.6 ± 2.6^f^	− 33.40 ± 0.32^ab^	0.21 ± 0.01^e^	90.03 ± 0.83^a^	22,670.4 ± 216.4^e^	3.10 ± 0.02^e^
0.19	332.3 ± 3.0^e^	− 33.70 ± 0.32^a^	0.41 ± 0.01^d^	90.15 ± 0.84^a^	24,732.7 ± 234.1^c^	3.20 ± 0.02^d^
0.21	401.5 ± 3.7^d^	− 32.10 ± 0.31^c^	0.53 ± 0.01^c^	84.20 ± 0.79^d^	25,082.6 ± 235.9^b^	3.40 ± 0.02^c^
0.23	483.1 ± 4.3^b^	− 31.40 ± 0.30^d^	0.64 ± 0.01^b^	78.13 ± 0.73^e^	26,439.8 ± 253.0^a^	4.00 ± 0.03^b^

Data represent mean ± standard deviations. Different letters in the same column represent a significant difference between samples (*p* < 0.05). Arrange all the averages in descending order, and use the letter “a” on the maximum average.

This mixture system lowers the interfacial tension and also prevents coalescence, and thus improves the stability of a protein-stabilized oil–water emulsion^33^. Therefore, after PC addition (from 0.13 to 0.19%), the ζ-potential value and the stability of the nanoemulsions were increased. Excessive PC addition increased the PC content in the aqueous phase of the emulsion. Based on the principle of displacement solubilisation, the oil–water interface protein was replaced by PC and entered the aqueous phase, which reduced the interfacial protein content and increased the particle size of the emulsion^35–37^.

### Effect of ultrasonic power

Ultrasonication is more economical and practical than microfluidization when taking into consideration production costs, maintenance, and aseptic production. Ultrasonication has become an excellent and superior tool in the emulsification process^38^. As shown in Table 3, an increase in ultrasonic power (from 100 to 400 W) led to an increase in average particle size, PDI, and TSI, but a decrease in turbidity value, which in turn increased ζ-potential and emulsion production. The cavitation effect produced by ultrasonication can reduce the particle size of SPI and affect the interfacial layer of the emulsion^39^. As ultrasonic power increased, the distribution became more uniform. These results suggest that 500 W resulted in the best values. After the ultrasonic power is increased to 500 W, the formation and collapse of cavitation bubbles can generate strong shock waves and jets. This strong force causes a significant increase in particle size and PDI that results in the tendency of the emulsion to re-agglomerate. Research has shown that the hydrophobicity of milk proteins was reported to have increased after ultrasonication^40,41^. The hydrophobic interaction forces aggregated droplets and increased the PDI, turbidity, and particle size of the emulsion. The increase in the of ultrasonic power increasesd the frequency of collisions between emulsion droplets, which subsequently leads to a higher probability of coalescence of droplets^28^. The increasing turbidity values may be related to the aggregation of emulsion molecules. The emulsification interface was unstable state under the condition of high ultrasonic power (600 W) output. At this time, the absolute value of the ζ-potential of the nanoemulsion was significantly reduced.

**Table 3:** Effect of ultrasonic power on the characteristics and stability of nanoemulsions.

Ultrasonic power (w)	Particle size (nm)	ζ-potential (mV)	PDI	Emulsification yield (%)	Turbidity	TSI
200	594.2 ± 5.5^a^	− 26.90 ± 0.21^e^	0.62 ± 0.01^a^	75.01 ± 0.72^d^	26,502.4 ± 250.1^a^	4.91 ± 0.04^a^
300	451.9 ± 4.0^b^	− 29.00 ± 0.24^c^	0.36 ± 0.01^c^	86.74 ± 0.84^c^	24,732.7 ± 235.0^c^	3.80 ± 0.03^c^
400	295.6 ± 2.4^c^	− 33.40 ± 0.29^b^	0.21 ± 0.01^d^	90.03 ± 0.87^ab^	24,659.0 ± 226.9^c^	3.10 ± 0.02^d^
500	291.8 ± 2.4^c^	− 34.30 ± 0.30^a^	0.22 ± 0.01^d^	91.04 ± 0.89^a^	23,702.1 ± 227.0^d^	3.01 ± 0.02^e^
600	479.7 ± 4.3^b^	− 28.40 ± 0.23^d^	0.51 ± 0.01^b^	74.95 ± 0.71^d^	25,018.1 ± 231.9^b^	3.90 ± 0.03^b^

Data represent mean ± standard deviations. Different letters in the same column represent a significant difference between samples (*p* < 0.05). Arrange all the averages in descending order, and use the letter “a” on the maximum average.

### Effect of ultrasonication time

The effect of ultrasonic processing time on the properties of nanoemulsions is shown in Table 4. PDI, turbidity, and the particle size of the nanoemulsions decreased and the emulsification yield increased when the ultrasound time increased from 6 to 9 min. Properly increasing the ultrasonic time can improve the dispersion state and emulsion stability of nanoemulsion. When the ultrasound time was continuously increased (between 9 and 10 min), the particle size increased from 287.9 to 468.5 nm. The particle size of the nanoemulsions increased by longer sonication treatment. The effect of long-term sonication on particle size is related referred to in the literature as ”over-processing”. Ultrasonication generated a large amount of energy, increasing the aggregation of droplets^34^. Lago et al. observed a similar trend with ultrasonic treatment of emulsion particle size^42^. The greater turbulence and heating of the system increased the frequency of collisions between the oil balls and caused emulsion instability^19^. Over-processing also caused the ζ-potential value to decrease^28,39,43^^.^ Additionally, previous researchers found that long-term ultrasonication decreased the emulsion stability due to a decrease in interfacial tension and viscosity, while smaller interfacial tension values caused emulsion interface instability^40,44^. Therefore, nanoemulsions exhibited satisfactory emulsion characteristics when the ultrasonic time was 9 min.

**Table 4:** Effect of ultrasonic power on the characteristics and stability of nanoemulsions.

Ultrasonic time (min)	Particle size (nm)	ζ-potential (mV)	PDI	Emulsification yield (%)	Turbidity	TSI
6	589.7 ± 5.4^a^	− 26.40 ± 0.19^b^	0.58 ± 0.01^a^	76.13 ± 0.74^c^	25,463.4 ± 247.3^a^	4.90 ± 0.04^a^
7	437.4 ± 4.0^b^	− 27.10 ± 0.21^b^	0.40 ± 0.01^b^	82.69 ± 0.80^b^	24,856.2 ± 235.7^b^	3.62 ± 0.03^b^
8	295.6 ± 2.4^c^	− 33.40 ± 0.29^a^	0.21 ± 0.00^c^	90.03 ± 0.87^a^	24,732.7 ± 234.4^b^	3.13 ± 0.02^c^
9	287.9 ± 2.4^c^	− 34.90 ± 0.31^a^	0.18 ± 0.00^c^	91.47 ± 0.90^a^	24,614.1 ± 238.3^b^	3.00 ± 0.02^c^
10	468.5 ± 4.1^b^	− 27.60 ± 0.23^b^	0.53 ± 0.01^a^	76.24 ± 0.74^c^	25,027.5 ± 243.1^a^	3.81 ± 0.03^a^

Data represent mean ± standard deviations. Different letters in the same column represent a significant difference between samples (*p* < 0.05). Arrange all the averages in descending order, and use the letter “a” on the maximum average.

### Ultrasonic nanoemulsion droplet structure

**Figure 1:**
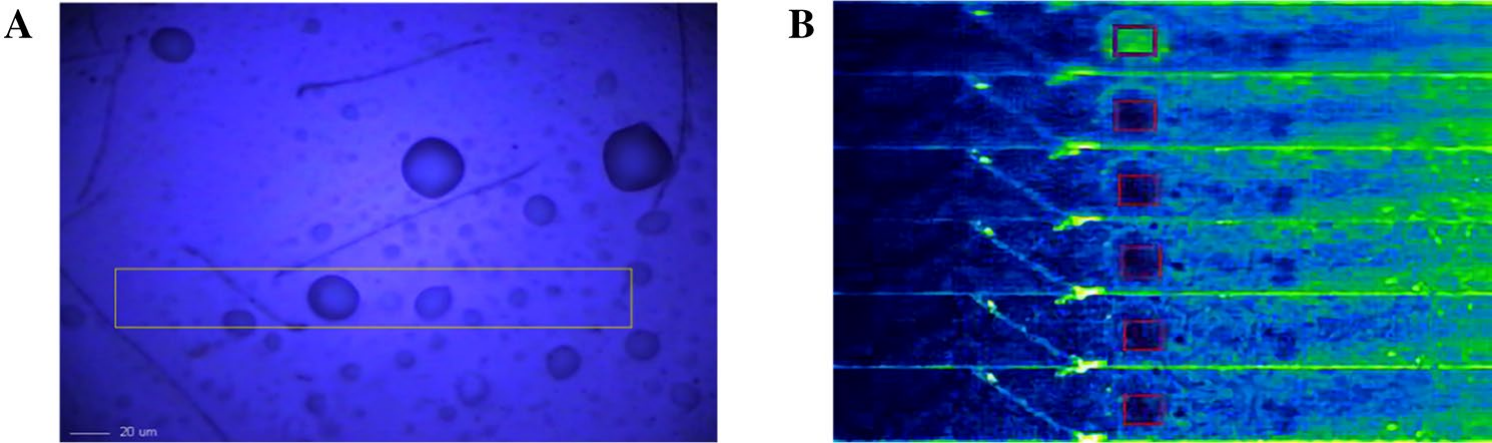
3D-Raman microscopic image of ultrasonic preparation of nanoemulsions. In the figure, (**A**) a single nanoemulsions droplet was selected for analysis, and (**B**) 6 Raman imaging plots of the single nanoemulsions droplet were formed as the observational displacement gradually probed into the oil phase.

The 3D images help to observe the spatial structural morphology. In this study, the 3D confocal Raman imaging was used to observe the structure of SPI-PC nanoemulsion. The protein in emulsion exhibited significant Raman absorption at 1,660 cm^−1^, and the feature regions were used for extraction and computational imaging. A microscopic image of a single drop in the yellow borderline region was analyzed in Fig. 1A. A Raman imaging structure of six nanoemulsion droplets (Fig. 1B) was formed as the observed position gradually probed into the center of the oil phase. The figure shows that a large number of green markers (SPI components) were more widely distributed in the oil–water interface of the emulsion droplets. It can be seen that soy protein were evenly distributed at the interface of nanoemulsion droplets according to our processing conditions.

**Figure 2:**
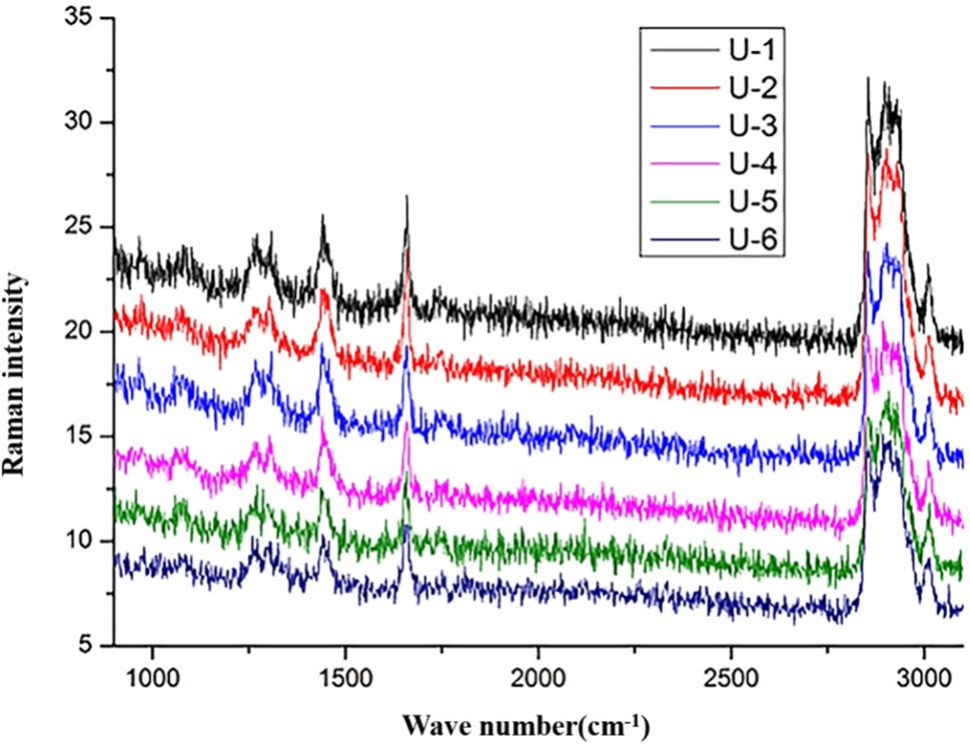
Raman image of the ultrasonic preparation of nanoemulsions. (U-1, U-2, U-3, U-4, U-5 and U-6 represent six observation positions from top to bottom, respectively).

Figure 2 shows the Raman characteristic absorption peak image of the emulsion. The symmetric stretching vibration and the anti-symmetric stretching vibration characteristic peak of the C–H_2_ group of PC were 2,840 cm^−1^ and 2,880 cm^−1^, respectively. The characteristic peak of amide I of SPI located at 1665 cm^−1^. The results indicated that PC and SPI were distributed at the oil–water interface of the emulsion droplets, and our results were consistent with those of a previous study^20^. The total Raman intensity decreased when the observed position gradually moved toward the center of the oil droplet, and this effect be related to the reduction of the soy protein content. However, the characteristic peaks of SPI and PC have not disappeared in the figure, which proved that SPI and PC were interacted and uniformly absorbed at the interface of the nanoemulsions.

The 3D images help to observe the spatial structural morphology. In this study, the 3D confocal Raman imaging was used to observe the structure of SPI-PC nanoemulsion. The protein in emulsion exhibited significant Raman absorption at 1,660 cm^−1^, and the feature regions were used for extraction and computational imaging. A microscopic image of a single drop in the yellow borderline region was analyzed in Fig. 1A. A Raman imaging structure of six nanoemulsion droplets (Fig. 1B) was formed as the observed position gradually probed into the center of the oil phase. The figure shows that a large number of green markers (SPI components) were more widely distributed in the oil–water interface of the emulsion droplets. It can be seen that soy protein were evenly distributed at the interface of nanoemulsion droplets according to our processing conditions.

Figure 2 shows the Raman characteristic absorption peak image of the emulsion. The symmetric stretching vibration and the anti-symmetric stretching vibration characteristic peak of the C-H_2_ group of PC were 2,840 cm^−1^ and 2,880 cm^−1^, respectively. The characteristic peak of amide I of SPI located at 1665 cm^−1^. The results indicated that PC and SPI were distributed at the oil–water interface of the emulsion droplets, and our results were consistent with those of a previous study^20^. The total Raman intensity decreased when the observed position gradually moved toward the center of the oil droplet, and this effect be related to the reduction of the soy protein content. However, the characteristic peaks of SPI and PC have not disappeared in the figure, which proved that SPI and PC were interacted and uniformly absorbed at the interface of the nanoemulsions.

### Storage stability of nanoemulsions

The stability of the product is essential for nanoemulsion-based delivery systems in most practical applications. Temperature, pH, storage time, processing method, and ionic strength can influence the stability of emulsions^20^. Storage temperature and time are main factors influencing the stability of emulsions. The effects of different storage temperatures and storage times on nanoemulsion stability is shown in Table 5, When the storage temperature increased, the particle size increased, and the β-carotene retention and ζ-potential rate decreased with both storage temperature and time. The average particle size of the emulsion was maintained at 100–500 nm of nanodroplets, and the ζ-potential of the nanoemulsion was reduced to 35–30 mV during storage. The rate of liquid phase separation of nanoemulsions during storage also depends on the frequency of contact collisions between droplets, because Brownian motion increases as temperature storage increases. The droplet collision frequency affected the mass transfer kinetics of surfactant and oil molecules between the water and oil phases, resulting in an increase of particle size^45^. The ζ-potential value was an important indicator for the stability of the nanoemulsions. After storage at 4 °C, 25 °C, and 55 °C for 30 days, the absolute ζ-potential values of emulsions gradually decreased to 31.5, 30.1, and 29.6, respectively. This may be due to the aging of austenite and the change in protein conformation over time, leading to the formation of hydrophobic bonds and hydrogen bonds between adjacent proteins at the interface^46^. Nanoemulsions had a lower electrostatic interaction while they were stored at high temperatures or during long-term storage, which caused the conformational change of the biopolymer of the surfactant molecules^47–49^.The stability nanoemulsion is related to the storage environment(time, temperature). The nanoemulsions gradually oxidized during storage, and the degree of oxidation increased with the increase in storage time and temperature. Lipid oxidation might change the interfacial composition of the nanoemulsion, and cause the emulsifier to rearrange and desorb at the interface and reduce the stability of the emulsion system. Eventually, the nanoemulsions gradually broke up, which caused β-carotene to be released from the nanoemulsions.

**Table 5:** Storage stability of nanoemulsion.

Sample	Storage time (d)	4 °C	25 °C	55 °C
Particle size (nm)	0	288.1 ± 1.92^f^	287.6 ± 1.51^f^	288.2 ± 2.18^e^
	5	290.4 ± 2.33^de^	292.1 ± 3.21^f^	338.2 ± 5.29^d^
	10	292.3 ± 2.21^cde^	302.1 ± 4.16^e^	344.7 ± 2.15^ cd^
	15	294.4 ± 3.35^ cd^	319.5 ± 2.21^d^	350.4 ± 4.14^c^
	20	297.3 ± 2.38^bc^	340.5 ± 5.19^c^	361.2 ± 6.19^b^
	25	300.2 ± 3.27^ab^	353.3 ± 5.26^b^	366.4 ± 3.15^ab^
	30	304.4 ± 4.29^a^	354.4 ± 5.28^a^	372.1 ± 4.23^a^
ζ-Potential (mV)	0	− 33.3 ± 0.13^a^	− 33.2 ± 0.22^a^	− 33.3 ± 0.16^a^
	5	− 33.1 ± 0.12^ab^	− 32.8 ± 0.29^b^	− 32.9 ± 0.23^b^
	10	− 32.9 ± 0.19^b^	− 32.4 ± 0.21^c^	− 32.5 ± 0.26^c^
	15	− 32.6 ± 0.15^c^	− 31.7 ± 0.24^d^	− 31.7 ± 0.27^d^
	20	− 32.2 ± 0.21^d^	− 31.2 ± 0.18^e^	− 30.9 ± 0.17^e^
	25	− 31.9 ± 0.16^e^	− 30.7 ± 0.13^f^	− 30.3 ± 0.25^f^
	30	− 31.5 ± 0.17^f^	− 30.1 ± 0.24^ g^	− 29.6 ± 0.23^ g^
β-Carotene retention rate (%)	0	98.5 ± 0.02^a^	98.5 ± 0.01^a^	98.5 ± 0.03^a^
	5	94.2 ± 0.23^b^	93.1 ± 0.31^b^	92.4 ± 0.31^b^
	10	92.6 ± 0.34^c^	91.7 ± 0.46^c^	90.2 ± 0.23^c^
	15	91.2 ± 0.32^d^	89.8 ± 0.51^d^	88.7 ± 0.46^d^
	20	89.3 ± 0.47^e^	88.5 ± 0.38^e^	87.5 ± 0.53^e^
	25	88.1 ± 0.19^f^	87.4 ± 0.27^f^	86.4 ± 0.32^f^
	30	87.8 ± 0.28^f^	87.1 ± 0.26^f^	86.1 ± 0.27^f^

Data represent mean ± standard deviations. Different letters in the same column represent a significant difference between samples (*p* < 0.05). Arrange all the averages in descending order, and use the letter “a” and “A” on the minimum average.

Although the retention rate of β-carotene in the emulsion gradually decreased, 86% of β-carotene was remained even storaging for a long time at a higher storage temperature (Table 5), exhibiting excellent resistance to droplet coalescence during storage. This may be due to the antioxidant activities of SPI and PC. Borba et al.^50^ demonstrated that Tween 20 β-carotene nanoemulsions prepared by a high-pressure homogenizer had a retention rate of 70–80% during storage, and an increased ability to encapsulate β-carotene as compared to conventional emulsions. Early studies have found that protein emulsifiers inhibit lipid oxidation better than small-molecule surfactants^51,52^. This provides a method to increase the potential use of carotenoids in the food industry, including applications of β-carotene.

## Conclusions

In this research, the SPI-PC complex was used as the aqueous phase and sunflower oil was used as the oil phase, and the β-carotene nanoemulsion was prepared using ultrasonic technology. The results show that when SPI is 1.5%, PC is 0.22%, ultrasonic power is 500 W, ultrasonic time is 9 min, nanoemulsion has the best stability. 3D Raman imaging shows that ultrasonic treatment can make SPI and PC evenly distributed on the water–oil interface of nanoemulsion droplets. During storage, the ζ-potential and β-carotene retention rate were higher than 30 mV and 86%, respectively. The optimal preparation process provides a method and theoretical basis for the ultrasonic preparation of nanoemulsions, and may lead to further food design, including the design of β-carotene.
